# A novel role of *Dermatophagoides farinae*-derived miR-276-3p in aggravating mite-induced allergic airway inflammation

**DOI:** 10.1128/spectrum.01923-25

**Published:** 2025-12-22

**Authors:** Xiao Zang, Shangde Jiang, Jinyan Yu, Lianzheng Ma, Wei Mei, Shanchao Hong, Wei Wang

**Affiliations:** 1 School of Public Health, Nanjing Medical University 12461, Nanjing, Jiangsu Province, China; 2 School of Clinical Laboratory Medicine, Nanjing Medical University 12461, Nanjing, Jiangsu Province, China; 3 Wuxi School of Medicine, Jiangnan University 66374, Wuxi, Jiangsu Province, China; 4 Department of Clinical Laboratory, Jiangnan University Medical Center, Wuxi, Jiangsu Province, China; 5 National Health Commission Key Laboratory on Parasitic Disease Prevention and Control, Jiangsu Provincial Key Laboratory on Parasites and Vector Control Technology, Jiangsu Institute of Parasitic Diseases 608817, Wuxi, Jiangsu Province, China; Hubei University of Medicine, Shiyan, China

**Keywords:** *Dermatophagoides farinae*, dfa-miR-276-3p, allergic airway inflammation, stanniocalcin 1, reactive oxygen species/nuclear factor kappa B, cross-kingdom regulation

## Abstract

*Dermatophagoides farinae* (DFA), the most prevalent aeroallergen in allergic asthma, releases extracellular vesicles (EVs) containing dfa-miR-276-3p, which plays an important role in DFA sensitization. In this study, we utilized AAV-dfa-miR-276-3p to create a mouse model with dfa-miR-276-3p overexpression in lungs. Using this model, we demonstrated that dfa-miR-276-3p acted as a priming factor that exacerbated DFA extract (DFE)-induced airway inflammation in mice. We also confirmed that dfa-miR-276-3p functioned as a priming pro-inflammatory factor in human bronchial epithelial BEAS-2B cells, enhancing the release of inflammatory cytokines induced by DFE. Furthermore, we found that dfa-miR-276-3p regulated stanniocalcin 1 (STC1) in a targeted manner, leading to increased inflammatory cytokine secretion and activation of the reactive oxygen species (ROS)/nuclear factor kappa B (NF-κB) pathway. Notably, the addition of recombinant human STC1 alleviated the inflammatory effects of dfa-miR-276-3p, reducing airway inflammation and dampening ROS/NF-κB signaling. Parallel findings in mouse models confirmed that dfa-miR-276-3p drove DFA-induced airway inflammation through STC1-dependent regulation of the ROS/NF-κB pathway. Our study reveals a cross-kingdom regulatory role for DFA-derived miRNAs in the pathogenesis of allergic asthma, highlighting dfa-miR-276-3p as a crucial priming factor in the process of allergic airway inflammation induced by DFA.

**IMPORTANCE:**

We demonstrated that dfa-miR-276-3p acted as a priming factor that exacerbated *Dermatophagoides farinae* (DFA) extract-induced airway inflammation in mice, and functioned as a priming pro-inflammatory factor in human bronchial epithelial BEAS-2B cells. In addition, dfa-miR-276-3p was found to regulate stanniocalcin 1 (STC1) in a targeted manner, leading to increased secretion of inflammatory cytokines and activation of the reactive oxygen species (ROS)/nuclear factor kappa B (NF-κB) pathway, and addition of recombinant human STC1 alleviated the inflammatory effects of dfa-miR-276-3p, reducing airway inflammation and dampening ROS/NF-κB signaling. Parallel findings in mouse models confirmed that dfa-miR-276-3p drove DFA-induced airway inflammation through STC1-dependent regulation of the ROS/NF-κB pathway. Our findings provide new insights into the role of DFA-derived miRNAs in the development of allergic asthma and propose an alternative pathway for DFA sensitization that may have significant clinical implications for allergy prevention and treatment.

## INTRODUCTION

*Dermatophagoides farinae* (DFA), a major species of house dust mites (HDMs), is one of the most prevalent aeroallergens associated with allergic asthma (1). Allergic asthma, which is characterized as an allergic airway inflammatory disease, often manifests during childhood (2). Remarkably, up to 76.84% of allergic asthma cases are linked to sensitivity to DFA in eastern China, underscoring its role as a significant trigger (3). While most studies focus on the protein components of DFA’s allergens, emerging evidence indicates that other sensitizers derived from DFA also contribute to HDM-related airway allergies (1). Further analysis of the allergenic components of DFA is crucial for fully understanding the mechanisms underlying HDM-induced allergic asthma in children and will offer valuable insights its prevention and treatment.

Extracellular vesicles (EVs) are small lipid membrane-bound particles released by nearly all cell types (4). These vesicles transport a variety of biomolecules, including proteins and nucleic acids (such as microRNAs), which are crucial for intercellular communication (4). Our previous studies successfully isolated EVs from DFA, demonstrating their significant allergenicity and immunogenicity (5). miRNA sequencing revealed similar expression profiles among DFA, DFA-derived EVs, and DFA culture supernatants, suggesting that EVs derived from DFA may serve as potential carriers for miRNA transport (6). Notably, when DFA-derived EVs were co-cultured with human bronchial epithelial BEAS-2B cells, we detected specific DFA miRNAs (dfa-miR-276-3p and dfa-novel-miR2) within the human cells (6). These findings provide compelling evidence that DFA-secreted EVs can deliver functional miRNAs into human airway epithelial cells, serving as effective vehicles for their action in the airway.

Growing evidence highlights the significant role of cross-kingdom regulation in host-pathogen interactions (7). For example, the gastrointestinal nematode *Heligmosomoides polygyrus* delivers small RNAs via EVs to mouse gut epithelial cells, thereby modulating host innate immunity (8). Similarly, plant-derived miRNAs, such as miR-162a found in larval food, influence honey bee caste development and developmental timelines (9, 10). In this context, dfa-miR-276-3p, which is abundant in DFA and its derived EVs and culture supernatants, has been linked to immune responses. Transcriptome analysis of airway epithelial cells BEAS-2B transfected with dfa-miR-276-3p revealed changes in inflammatory pathways (6), suggesting that dfa-miR-276-3p has cross-kingdom regulatory capabilities and may play a role in shaping human immune responses to DFA sensitization.

Due to their lipid bilayers and nanoscale size, EVs can be easily phagocytosed and internalized by various cell types (11). Since EVs derived from DFA are likely to reach and penetrate airway epithelial cells more quickly than other allergenic components, we hypothesize that EV-packaged dfa-miR-276-3p is secreted into the environment and subsequently inhaled into the respiratory tract. As the ancient Chinese proverb states, “Prepare the supplies before sending troops,” we propose that EV-packaged dfa-miR-276-3p acts as the “provision” in allergic sensitization, priming the airway before exposure to other allergenic components. This early-phase mechanism allows dfa-miR-276-3p to serve as a priming mediator in DFA-induced sensitization, orchestrating the cross-kingdom regulation of human allergic airway inflammation.

To test our hypothesis, we first overexpressed dfa-miR-276-3p in mouse lung and airway epithelial cells. We then constructed allergic airway inflammation models using DFA extract in both animal and cellular systems with dfa-miR-276-3p overexpression to observe changes in inflammation-related indicators. This study enabled us to investigate the priming role of dfa-miR-276-3p in DFA-induced sensitization and to elucidate the underlying cross-kingdom regulatory mechanisms.

## MATERIALS AND METHODS

### Cell culture and transfection

Human bronchial epithelial BEAS-2B cells were purchased from Procell Biological Company (Shanghai, China), and cultured in Dulbecco’s modified Eagle’s medium (Gibco, Carlsbad, CA, USA) supplemented with 10% fetal bovine serum (Gibco) and 1% penicillin-streptomycin (Gibco) in the thermostat incubator at 37°C containing 5% CO_2_. When growing to 40% confluence, BEAS-2B cells were transfected with dfa-miR-276-3p mimic (RiboBio, Guangzhou, China) or mimic NC using the riboFECT CP Transfection Kit (RiboBio) following the manufacturer’s instructions.

### Modeling allergic airway inflammation in mice

Female 5-week-old C57BL/6 mice were obtained from the Center for Laboratory Animals, Yangzhou University and housed in the Laboratory Animal Center of Jiangsu Institute of Parasitic Diseases in a specific pathogen-free environment at 21–25°C, 65% humidity, 10–14 h light-dark cycle. The mice were anesthetized with isoflurane and then intranasally administered an adeno-associated virus serotype 9 (AAV9) carrying dfa-miR-276-3p (AAV-dfa-miR-276-3p; Zebrafish, Nanjing, China) to overexpress dfa-miR-276-3p in the airways. AAV9-negative control (AAV-NC) was used as a negative control. On day 21, sensitization was performed via intranasal delivery of 100 μg *D. farinae* extract (DFE; Greer Laboratories, Lenoir, NC, USA). From days 28 to 32, mice received daily intranasal challenges with 10 μg DFE to establish allergic inflammation. All mice were sacrificed on day 35 for analysis.

### Dust sample collection

Dust samples were collected from floors, carpets, couches, air conditioners, curtains, and bedding of inhabited environments using a vacuum cleaner. Then, 5 g of house dust samples were suspended in 40 mL of phosphate-buffered saline (PBS), stirred for 4 h, and then placed at 4°C for 4 h as previously described (6). The supernatant was obtained for the subsequent PCR verification of DFA and quantitative real-time PCR (qPCR) analysis of dfa-miR-276-3p expression.

### PCR assay

Genomic DNA was isolated from dust samples using the tissue DNA extraction kit (Magnetic Beads Method) (Wuxi MedTech Biomedicine Co., Ltd., Wuxi, China). Then, DFA was verified in dust samples using a nested PCR assay as described previously (12), and specific primers are provided in Table S1.

### qPCR assay

Total RNA was extracted using the FastPure Cell/Tissue Total RNA Isolation Kit V2 (Vazyme, Nanjing, China). The mRNAs and miRNAs were reversely transcribed to cDNA using the HiScript IV All-in-One Ultra RT SuperMix for qPCR (Vazyme) and the Bulge-Loop miRNA qRT-PCR Starter Kit (RiboBio), and the obtained cDNA was amplified with the ChamQ Universal SYBR qPCR Master Mix (Vazyme) on the LightCycler 480 RT-QPCR System (Roche, Rotkreuz, Switzerland). The dfa-miR-276-3p expression was quantified by a qPCR assay with specific primers shown in Table S2 with β-actin, U6, or cel-miR-39-3p as internal controls. The relative gene expression levels were analyzed using the 2^−∆∆C*t*^ method.

### Enzyme-linked immunosorbent assay (ELISA)

The concentrations of interleukin (IL)-6 (Absin, Shanghai, China), IL-33 (4A Biotech, Suzhou, China) and thymic stromal lymphopoietin (TSLP; 4A Biotech) were measured in the cell culture supernatant using commercial ELISA kits following the manufacturer’s instructions, and the concentrations of IL-4, IL-5 (4A Biotech), and IL-10 (Mlbio, Shanghai, China) were measured in bronchoalveolar lavage fluid (BALF). In addition, the total IgG level (Mlbio) was detected in mouse serum samples, and the stanniocalcin 1 (STC1) level was measured in human serum samples and cell culture supernatants using the commercial ELISA kits (Cloud-clone Corporation, Wuhan, China).

### CCK-8 assay

BEAS-2B cells were seeded in 96-well plates for 12 h. Cells were transfected with mimic NC and dfa-miR-276-3p mimic for 24 h, followed by treatment with 50 μg/mL DFE for 24 h, while the PBS group was exposed to an equal volume of PBS. Cell viability was measured using the CCK-8 assay (Beyotime, Shanghai, China).

### Flow cytometry

BEAS-2B cells were seeded onto 24-well plates and treated as described above. Then, 5 μL of Annexin V-FITC (MeilunBio, Dalian, China) and 10 μL of PI (MeilunBio) were added to the cell suspension to incubate together for 15 min at room temperature. Following incubation, 400 μL of binding buffer was added into each tube. Subsequently, cell apoptosis was detected using a flow cytometer (Beckman Coulter; Brea, CA, USA) and analyzed using the FlowJo 10.8.1 software.

### Dual-luciferase report assay

The 3′-UTR of STC1, containing the predicted dfa-miR-276-3p seed-matching site, was amplified from the cDNA library using PCR assay. The wild-type and mutant 3′-UTR sequences of STC1 mRNA were cloned into the pmiR-REPORT-3′-UTR vectors containing the luciferase sequence to construct reporter plasmids. Subsequently, STC1-WT (wild-type 3′-UTR) or STC1-Mut (mutant 3′-UTR) and dfa-miR-276-3p mimic or mimic NC were co-transfected into BEAS-2B cells for 48 h, and then subjected to dual-luciferase reporter assay with the Dual Luciferase Reporter Gene Assay Kit (Yeasen, Shanghai, China). Luciferase activity was determined with the Spark multimode microplate reader (TECAN, Zürich, Switzerland).

### Serum samples collection

Serum samples were collected from 50 children with allergic airway inflammation (age range, 1–14 years, with a mean age of 6.98 years) who were allergic to DFA, and 50 healthy children (age range, 1–14 years, with a mean age of 6 years). Detailed demographic and clinical characteristics of the participants are presented in Table S3. The expression level of DFA-specific IgE antibody was measured using the CAP System (Pharmacia & Upjohn Diagnostics AB, Bridgewater, NJ, USA).

### Western blotting

Total protein was separated using 12.5% sodium dodecyl sulfate polyacrylamide gel electrophoresis (Epizyme, Shanghai, China), and the blots were transferred to 0.45 μm polyvinylidene fluoride membranes (Millipore, Burlington, MA, USA) and blocked with 5% skim milk (Beyotime). Then, the membranes were incubated at 4°C overnight with phospho-nuclear factor kappa B (p-NF-κB) p65 (Cell Signaling Technology, Inc., Danvers, MA, USA), NF-κB p65 (Cell Signaling Technology, Inc.), and STC1 (Gentex, Zeeland, MI, USA) primary antibodies, while β-actin (Bioss, Beijing, China) served as a loading control. Subsequently, the immunoblots were incubated with the secondary HRP-conjugated goat anti-rabbit IgG antibody (Bioss). The bands were visualized using the ECL Chemiluminescence Kit (Vazyme) and the ImageQuant 800 imaging system (Cytiva, Uppsala, Sweden).

### Immunofluorescence

Mouse lung tissue sections or cells were fixed in 4% paraformaldehyde (Biosharp, Beijing, China). Then, cells were permeabilized with Triton X-100 (Beyotime). Sections or cell culture slides were blocked with 10% goat serum at room temperature, and the cell culture slides were incubated with the primary anti-NF-κB p65 antibody (Cell Signaling Technology, Inc.), while the lung tissue sections were incubated with the primary anti-claudin 1 antibody (Proteintech, Manchester, UK). Subsequently, sections or slides were incubated in the secondary CY3-Conjugated Goat Anti-rabbit IgG (H + L) antibody (Boster, Wuhan, China) at room temperature. Cell slides were then stained with DAPI (Beyotime). Images were visualized under the Zeiss Observer 7 (Zeiss, Göttingen, Germany).

### Reactive oxygen species (ROS) detection

BEAS-2B cells or homogenized mouse lung tissues were incubated with 2′,7′-Dichlorofluorescin diacetate (DCFH-DA, Beyotime) or DHE probes (Biolab, Wuhan, China), respectively. Fluorescence intensity was imaged with the Olympus BX63 fluorescence microscope (Olympus, Tokyo, Japan) or assessed with the Spark multimode microplate reader (TECAN, Morrisville, NC, USA), normalized to protein content.

### Statistical analysis

All data were presented as mean ± standard error of the mean (SEM) and analyzed using GraphPad Prism 9 and ImageJ 2.0.0. Difference of means among groups was tested for statistical significance with one-way analysis of variance (ANOVA), followed by Tukey’s post hoc test. A *P* value of <0.05 was considered statistically significant.

## RESULTS

### Identification of DFA and dfa-miR-276-3p in inhabited environments

**Fig 1:**
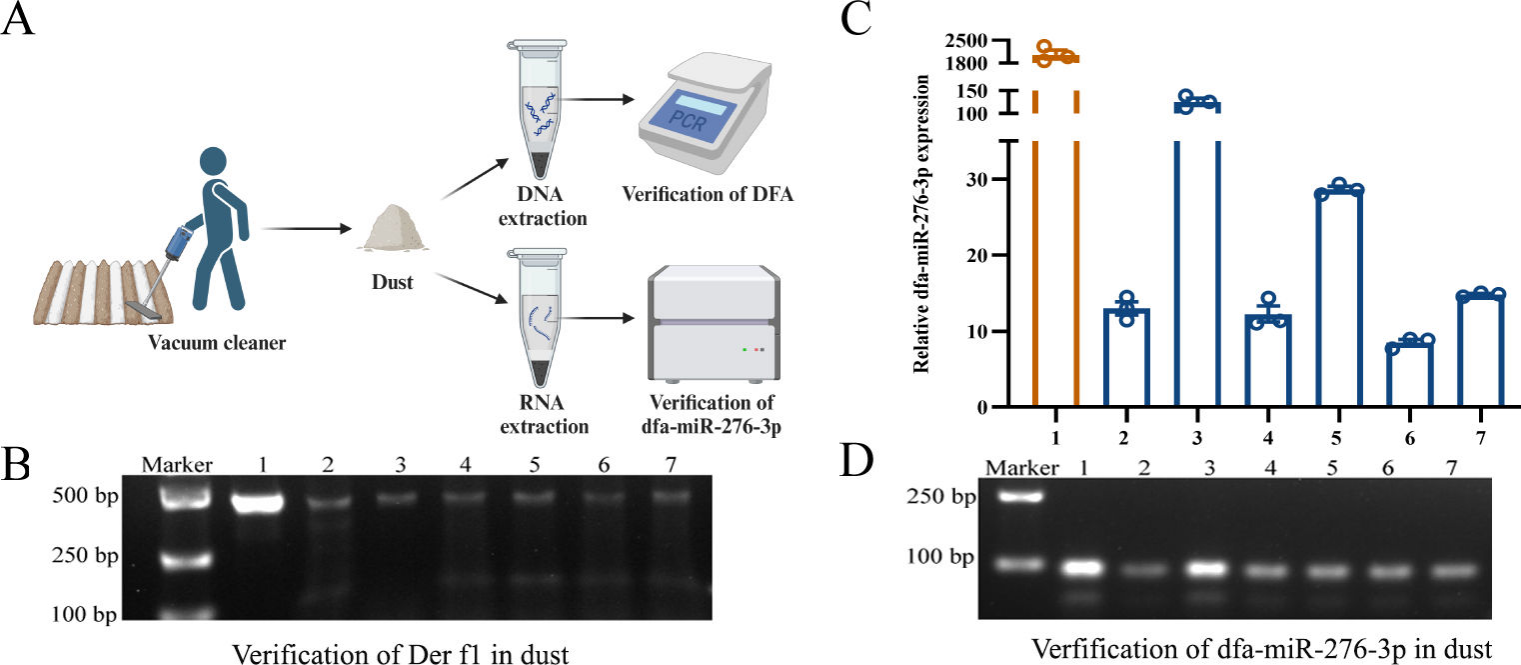
Identification of DFA and dfa-miR-276-3p in inhabited environments. (**A**) Workflow diagram for different treatments of dust samples. (**B**) PCR assay confirms presence of DFA Der f1 in inhabited dust samples. (**C**) qPCR assay verifies presence of dfa-miR-276-3p in inhabited dust samples. (**D**) Electrophoresis detects the presence of dfa-miR-276-3p in inhabited dust samples. In images (**B–D**), lane 1 shows the DFA template, used as a positive control, while lanes 2–7 represent dust samples collected from floors, carpets, couches, air conditioners, curtains, and bedding. Data are presented as means ± SEM.

To confirm whether DFA carries dfa-miR-276-3p into external environments, we collected dust samples for PCR and qPCR assays to verify the presence of both DFA and dfa-miR-276-3p in inhabited spaces (Fig. 1A). Derf 1 is the primary allergenic component of DFA, and detection of *Derf 1* gene in dust samples confirms the presence of DFA. In this study, *Derf 1* gene was detected in dust samples collected from floors, carpets, couches, air conditioners, curtains, and bedding in residential environments (Fig. 1B). Additionally, qPCR assays identified dfa-miR-276-3p in dust samples (Fig. 1C), and agarose gel electrophoresis confirmed its presence (Fig. 1D). These findings confirm the presence of DFA and dfa-miR-276-3p in inhabited environments, providing a foundation for further investigations into the role of dfa-miR-276-3p in DFA sensitization.

### Dfa-miR-276-3p as a prime exacerbates DFE-induced airway inflammation *in vivo*

**Fig 2:**
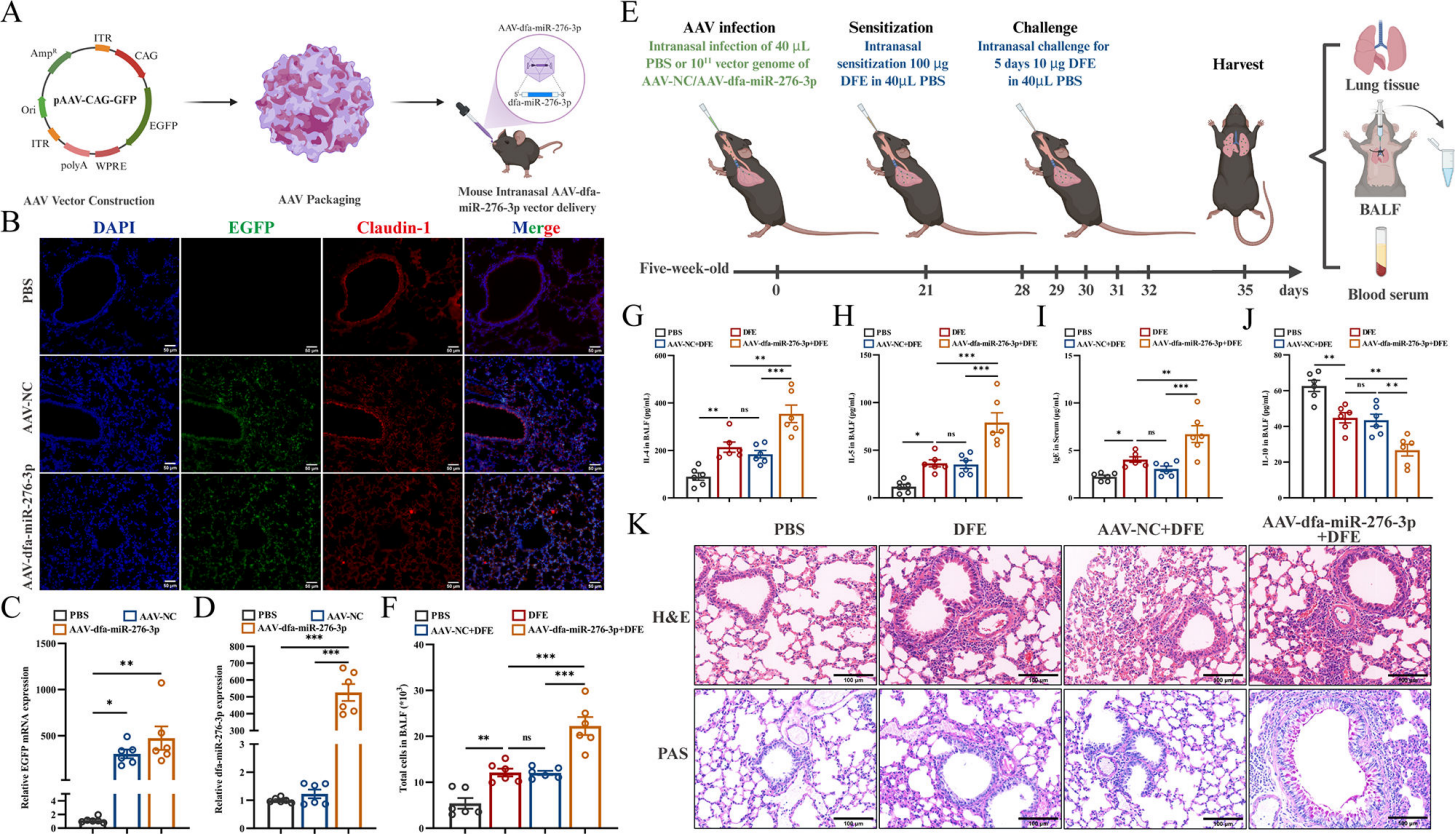
Dfa-miR-276-3p as a prime exacerbates DFE-induced airway inflammation *in vivo*. (**A**) Schematic diagram for construction of AAV-dfa-miR-276-3p-overexpressing mouse models. (**B**) Immunofluorescence assay detects co-expression of EGFP (green) and Claudin-1 (red) in mouse lung tissues, and the nuclei are stained with DAPI (blue) (*n* = 3). Scale bar = 50 μm. (**C**) qPCR assay quantifies EGFP mRNA expression in mouse lung tissues. (**D**) qPCR assay quantifies dfa-miR-276-3p expression in mouse lung tissues. (**E**) Schematic diagram for construction of a mouse model of airway sensitization following AAV-dfa-miR-276-3p overexpression in lungs. (**F**) Total cell counts in mouse BALF (*n* = 3). (**G**) ELISA measures IL-4 levels in mouse BALF (*n* = 3). (**H**) ELISA measures IL-5 levels in mouse BALF (*n* = 3). (**I**) ELISA measures IgE antibody levels in mouse serum samples (*n* = 6). (**J**) ELISA measures IL-10 levels in mouse BALF (*n* = 3). (**K**) HE and PAS staining of representative lung sections (*n* = 3). Scale bar = 100 μm. ns, not significant, *P < 0.05, **P < 0.01, and ***P < 0.001. Data are presented as means ± SEM. Difference of means among groups is tested for statistical significance with ANOVA, followed by Tukey’s post hoc test.

To investigate the *in vivo* priming effect of dfa-miR-276-3p on DFE-induced airway inflammation, we generated a lung-specific overexpression mouse model using intranasal delivery of AAV-dfa-miR-276-3p (Fig. 2A). Successful transduction was confirmed through qPCR and immunofluorescence, which detected both dfa-miR-276-3p and enhanced green fluorescent protein (EGFP) in lung tissues (Fig. 2B through D). Following DFE sensitization (Fig. 2E), the AAV-dfa-miR-276-3P + DFE group exhibited significantly increased total cell counts and pro-inflammatory cytokines (IL-4 and IL-5) in BALF compared to the DFE-only and AAV-NC + DFE control groups (Fig. 2 through H). Additionally, serum IgE levels were elevated (Fig. 2I), while anti-inflammatory IL-10 production was reduced (Fig. 2J). Furthermore, histological analysis revealed increased inflammatory cell infiltration and mucus secretion in lung tissues (Fig. 2K). These findings demonstrate that pulmonary pretreatment with dfa-miR-276-3p exacerbates DFE-induced airway inflammation *in vivo*.

### Pretreatment with dfa-miR-276-3p aggravates DFE-triggered inflammation *in vitro*

**Fig 3:**
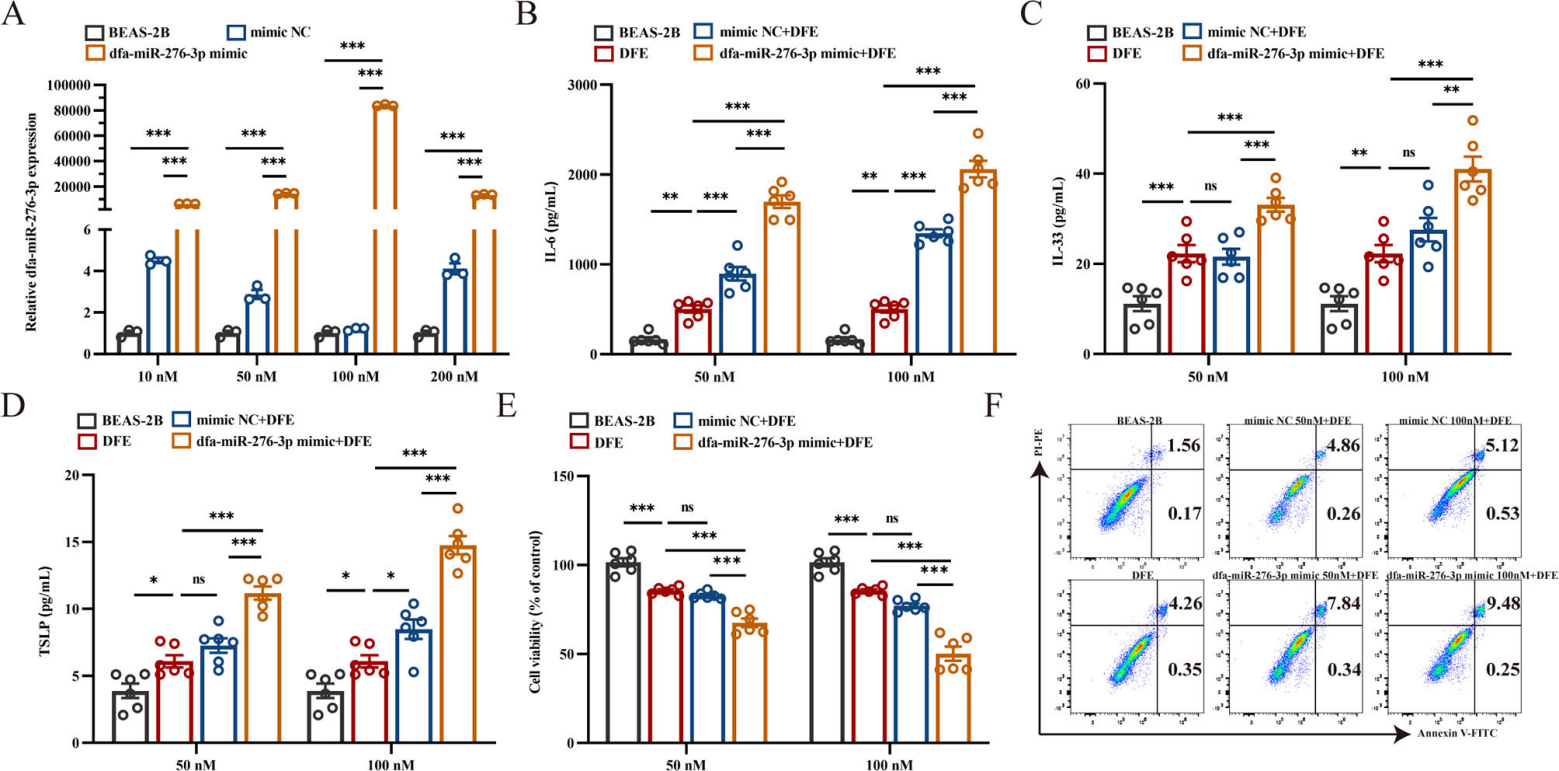
Pretreatment with dfa-miR-276-3p aggravates DFE-triggered inflammation *in vitro*. (**A**) qPCR quantifies dfa-miR-276-3p expression in BEAS-2B cells (*n* = 3). (**B**) ELISA measures IL-6 levels in the culture supernatant of BEAS-2B cells (*n* = 6). (**C**) ELISA measures IL-33 levels in the culture supernatant of BEAS-2B cells (*n* = 6). (**D**) ELISA measures TSLP levels in the culture supernatant of BEAS-2B cells (*n* = 6). (**E**) CCK-8 assay measures the viability of BEAS-2B cells (*n* = 6). (**F**) Flow cytometry detects the apoptosis of BEAS-2B cells (*n* = 3). ns, not significant, *P < 0.05, **P < 0.01, and ***P < 0.001. Data are presented as means ± SEM. Difference of means among groups is tested for statistical significance with ANOVA, followed by Tukey’s post hoc test.

To further explore the *in vitro* priming effect of dfa-miR-276-3p on DFE-induced inflammation, BEAS-2B cells were pretreated with a dfa-miR-276-3p mimic prior to DFE exposure. Transfection efficiency was assessed using qPCR, which revealed optimal uptake at concentrations of 50 and 100 nM (Fig. 3A), and these concentrations were selected for subsequent experiments. To evaluate the role of dfa-miR-276-3p in DFE-induced inflammation, we measured the secretion of pro-inflammatory cytokines IL-6, IL-33, and TSLP. Compared to DFE treatment alone, BEAS-2B cells overexpressing dfa-miR-276-3p exhibited significantly elevated cytokine levels following DFE exposure (Fig. 3B through D). Additionally, pretreatment with dfa-miR-276-3p mimic exacerbated DFE-induced cytotoxicity, resulting in reduced cell viability and increased apoptosis (Fig. 3E and F). These findings demonstrate that dfa-miR-276-3p functions as a pro-inflammatory enhancer, aggravating DFE-induced inflammation *in vitro*.

### Identification of STC1 as a target of dfa-miR-276-3p and its role in alleviating pro-inflammatory responses in BEAS-2B cells with recombinant human STC1 (rhSTC1) supplementation

**Fig 4:**
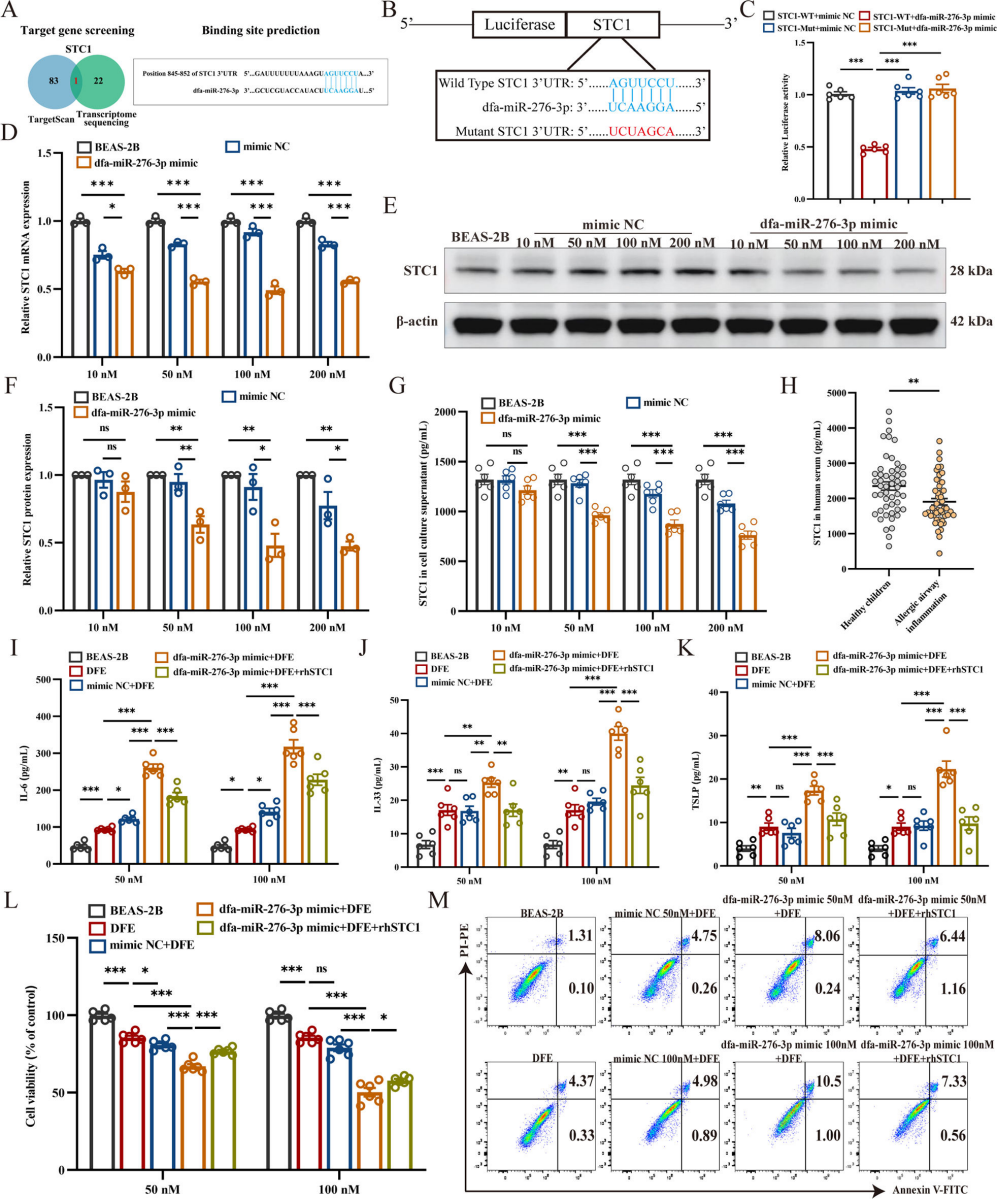
Identification of STC1 as a target of dfa-miR-276-3p and its role in alleviating pro-inflammatory responses in BEAS-2B cells with rhSTC1 supplementation. (**A**) Prediction of the targeting mRNA and potential binding site (blue) of dfa-miR-276-3p based on TargetScan and transcriptome sequencing. (**B**) Construction of luciferase reporter plasmids for STC1 with wild-type (blue) and mutant (red) 3′UTR sequences. (**C**) Luciferase activity in BEAS-2B cells following co-transfection with dfa-miR-276-3p mimic/mimic NC and STC1-WT or STC1-Mut (*n* = 6). (**D**) qPCR assay quantifies STC1 mRNA expression in BEAS-2B cells (*n* = 3). (**E and F**) Western blotting assay determines the STC1 protein expression in BEAS-2B cells (*n* = 3). (**G**) ELISA measures STC1 levels in the culture supernatant of BEAS-2B cells (*n* = 6). (**H**) ELISA measures STC1 levels in serum samples from healthy children (*n* = 50) and children with DFA-associated allergic airway inflammation (*n* = 50). (**I–K**) ELISA measures IL-6, IL-33, and TSLP levels in the culture supernatant of BEAS-2B cells (*n* = 6). (**L**) CCK-8 assay measures the viability of BEAS-2B cells (*n* = 6). (**M**) Flow cytometry detects the apoptosis of BEAS-2B cells (*n* = 3). ns, not significant, *P < 0.05, **P < 0.01, and ***P < 0.001. Data are presented as means ± SEM. Difference of means among groups is tested for statistical significance with ANOVA, followed by Tukey’s post hoc test.

In our previous study, we overexpressed dfa-miR-276-3p in BEAS-2B cells and conducted transcriptome sequencing, which identified 22 differentially expressed genes (DEGs) (fold change > 2, *Q* < 0.05) (6). These data have been deposited in the NCBI BioProject database (accession number: PRJNA1023698). Additionally, we utilized the TargetScan database to predict potential target genes of dfa-miR-276-3p, identifying 83 candidate genes. By integrating these data sets, we found that STC1 was the overlapping gene (Fig. 4A). To validate the direct regulatory interaction between dfa-miR-276-3p and STC1, we performed a dual-luciferase reporter assay (Fig. 4B). BEAS-2B cells co-transfected with the STC1-wild type (STC1-WT) reporter plasmid and the dfa-miR-276-3p mimic exhibited significantly reduced luciferase activity compared to cells transfected with either the STC1-mutant (STC1-Mut) reporter or the mimic-NC (negative control) (Fig. 4C). These results confirm that dfa-miR-276-3p directly targets STC1.

To further explore the regulatory role of dfa-miR-276-3p on STC1, we transfected BEAS-2B cells with the dfa-miR-276-3p mimic and observed a significant reduction in STC1 mRNA levels through qPCR analysis (Fig. 4D). Consistent with these findings, both the intracellular production and extracellular secretion of STC1 protein were markedly decreased following transfection with the dfa-miR-276-3p mimic (Fig. 4E through G). To assess the clinical relevance of this regulatory mechanism, we analyzed STC1 expression in serum samples from healthy children and those with DFA-associated allergic asthma. Notably, STC1 levels were significantly lower in the serum of children with DFA-associated allergic asthma compared to healthy controls (Fig. 4H). Collectively, these results demonstrate that dfa-miR-276-3p directly downregulates STC1 expression in BEAS-2B cells and that STC1 levels are consistently reduced in the serum of children with DFA-associated allergic asthma, suggesting its potential involvement in the pathogenesis of allergic asthma.

To further investigate the pro-inflammatory effects of dfa-miR-276-3p mediated by STC1, BEAS-2B cells were transfected with the dfa-miR-276-3p mimic and subsequently treated with rhSTC1. Compared to BEAS-2B cells treated with DFE alone after transfection, the addition of rhSTC1 significantly decreased the secretion of inflammatory cytokines IL-6, IL-33, and TSLP (Fig. 4I through K). Furthermore, rhSTC1 treatment alleviated the negative effects of dfa-miR-276-3p on cell viability and apoptosis (Fig. 4L and M). These results indicate that restoring rhSTC1 expression mitigates DFE-induced inflammation in BEAS-2B cells, which is exacerbated by dfa-miR-276-3p.

### Dfa-miR-276-3p exacerbates inflammation by suppressing STC1 to activate the ROS/NF-κB pathway

NF-κB and ROS are well-established mediators of inflammatory pathogenesis in various diseases (13, 14). STC1 has been shown to mitigate oxidative stress by reducing ROS levels (15) and to suppress inflammation by inhibiting the ROS/NF-κB signaling pathway during depression and renal ischemia-reperfusion injury (16, 17). Based on these findings, we investigated whether dfa-miR-276-3p exacerbates DFA-sensitized airway inflammation by targeting STC1 and dysregulating the ROS/NF-κB pathway.

**Fig 5:**
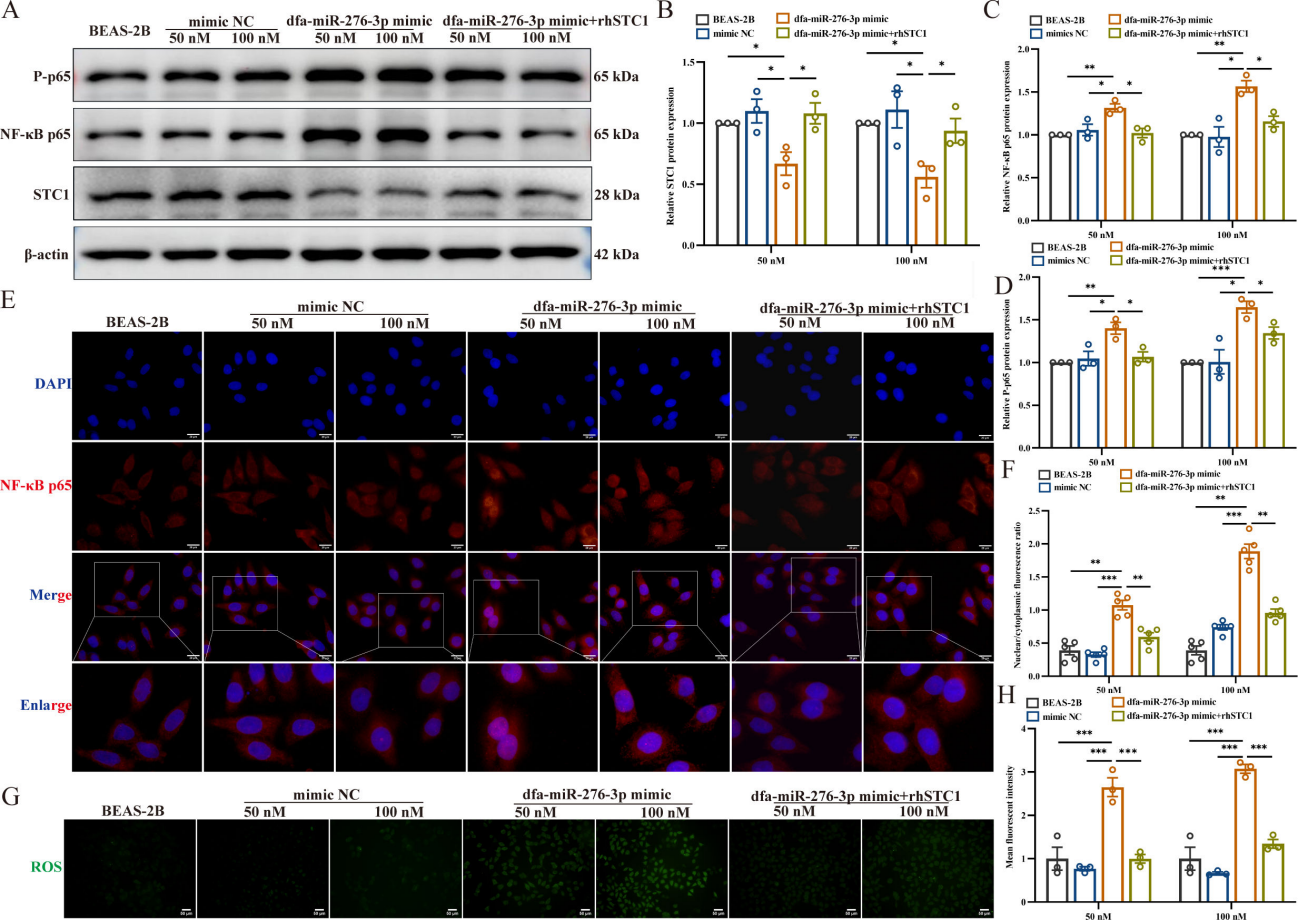
Dfa-miR-276-3p exacerbates inflammation by suppressing STC1 to activate the ROS/NF-κB pathway. (**A–D**) Western blotting assay determines the STC1, NF-κB p65, and p-p65 protein expression in BEAS-2B cells post-transfection with dfa-miR-276-3p and with addition of rhSTC1 (*n* = 3). (**E**) Representative immunofluorescence images of nuclear translocation of NF-κB p65 (red) in BEAS-2B cells post-transfection with dfa-miR-276-3p and with addition of rhSTC1, and the nuclei are stained with DAPI (blue) (*n* = 3). Scale bar = 20 μm. (**F**) Nuclear translocation of NF-κB p65 is quantified and indicated as a ratio of nuclear to cytoplasmic fluorescence (*n* = 5). (**G**) Representative fluorescence microscopic images of intracellular ROS stained with DCFH-DA (*n* = 3). Scale bar = 50 μm. (**H**) Densitometric fluorescence intensity of ROS (*n* = 3). ns, not significant, *P < 0.05, **P < 0.01, and ***P < 0.001. Data are presented as means ± SEM. Difference of means among groups is tested for statistical significance with ANOVA, followed by Tukey’s post hoc test.

To elucidate the role of dfa-miR-276-3p in modulating the ROS/NF-κB pathway through STC1 suppression, we analyzed the expression of STC1, NF-κB p65, and phosphorylated p65 (p-p65), as well as NF-κB nuclear translocation and intracellular ROS levels in BEAS-2B cells transfected with the dfa-miR-276-3p mimic. Western blotting analysis revealed that overexpression of dfa-miR-276-3p significantly downregulated STC1 while upregulating NF-κB p65 and p-p65 (Fig. 5A through D). Consistent with these findings, immunofluorescence assays showed enhanced nuclear translocation of NF-κB p65 (Fig. 5E and F) and a marked increase in ROS production (Fig. 5G and H). It is worth noting that rhSTC1 treatment reversed the reduction in STC1 levels, decreased NF-κB activation, reduced NF-κB nuclear translocation, and lowered ROS accumulation. These results collectively indicate that dfa-miR-276-3p exacerbates oxidative stress and inflammatory signaling by targeting STC1, thereby activating the ROS/NF-κB axis, a mechanism likely contributing to DFA-induced allergic airway inflammation.

### Dfa-miR-276-3p promotes DFE-induced airway inflammation through inhibiting STC1 expression to regulate ROS/NF-κB pathway *in vivo*

**Fig 6:**
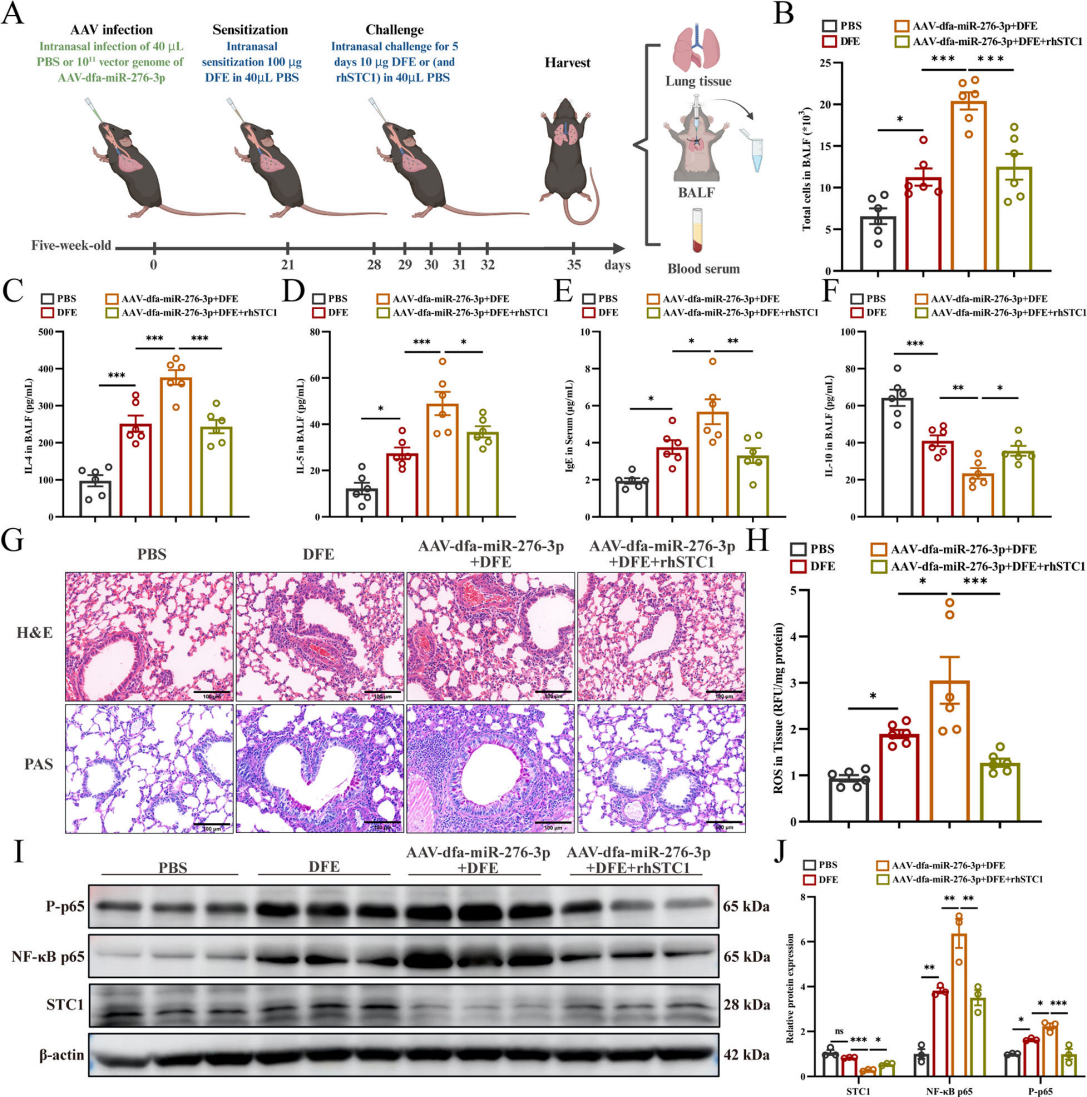
Dfa-miR-276-3p promotes DFE-induced airway inflammation through inhibiting STC1 expression to regulate ROS/NF-κB pathway *in vivo*. (**A**) Schematic diagram for different treatments of mice. (**B**) Total cell counts in mouse BALF (*n* = 3). (**C**) ELISA measures IL-4 levels in mouse BALF (*n* = 3). (**D**) ELISA measures IL-5 levels in mouse BALF (*n* = 3). (**E**) ELISA measures IgE antibody levels in mouse serum samples (*n* = 6). (**F**) ELISA measures IL-10 levels in mouse BALF (*n* = 3). (**G**) HE and PAS staining of representative lung sections (*n* = 3). Scale bar = 100 μm. (**H**) ROS levels in mouse lung tissues (*n* = 6). (**I and J**) Western blotting determines STC1, NF-κB p65, and p-p65 protein expression in mouse lung tissues (*n* = 3). ns, not significant, *P < 0.05, **P < 0.01, and ***P < 0.001. Data are presented as means ± SEM. Difference of means among groups was tested for statistical significance with ANOVA, followed by Tukey’s post hoc test.

To investigate the mechanism underlying the contribution of dfa-miR-276-3p to DFE-induced airway inflammation, mice were divided into four groups, including the PBS control group, the DFE-sensitized group, the AAV-dfa-miR-276-3P + DFE group, and the AAV-dfa-miR-276-3P + DFE + rhSTC1 group, following the sensitization protocol outlined in Fig. 6A. Compared to the DFE-sensitized group, the AAV-dfa-miR-276-3P + DFE group exhibited significantly elevated total cell counts and increased secretion of pro-inflammatory cytokines IL-4 and IL-5 in BALF, along with higher serum IgE levels (Fig. 6B through E). In contrast, the AAV-dfa-miR-276-3P + DFE + rhSTC1 group showed a reduction in these inflammatory markers. Importantly, levels of the anti-inflammatory cytokine IL-10, which were markedly suppressed in the AAV-dfa-miR-276-3P + DFE group, were restored with rhSTC1 co-treatment (Fig. 6F). Histopathological analysis revealed exacerbated inflammatory cell infiltration and mucus hypersecretion in mouse lungs of the AAV-dfa-miR-276-3P + DFE group, both of which were significantly alleviated by rhSTC1 administration (Fig. 6G).

At the molecular level, treatment with AAV-dfa-miR-276-3P + DFE significantly increased ROS production in mouse lung tissues compared to treatment with DFE alone, an effect that was mitigated by rhSTC1 supplementation (Fig. 6H). Western blotting analysis confirmed activation of the NF-κB pathway, with increased levels of NF-κB p65 and p-p65 in the AAV-dfa-miR-276-3P + DFE group. Notably, rhSTC1 co-treatment effectively suppressed both NF-κB p65 and p-p65 levels (Fig. 6I and J).

In summary, these findings indicate that dfa-miR-276-3p exacerbates DFE-induced allergic airway inflammation by inhibiting STC1 expression, which activates the ROS/NF-κB signaling axis, leading to enhanced pro-inflammatory responses and oxidative stress. Conversely, rhSTC1 effectively counteracts these pathological effects, restoring a more balanced inflammatory response. Thus, dfa-miR-276-3p acts as an upstream regulator that influences allergic airway inflammation *in vivo* by modulating STC1 expression.

## DISCUSSION

**Fig 7:**
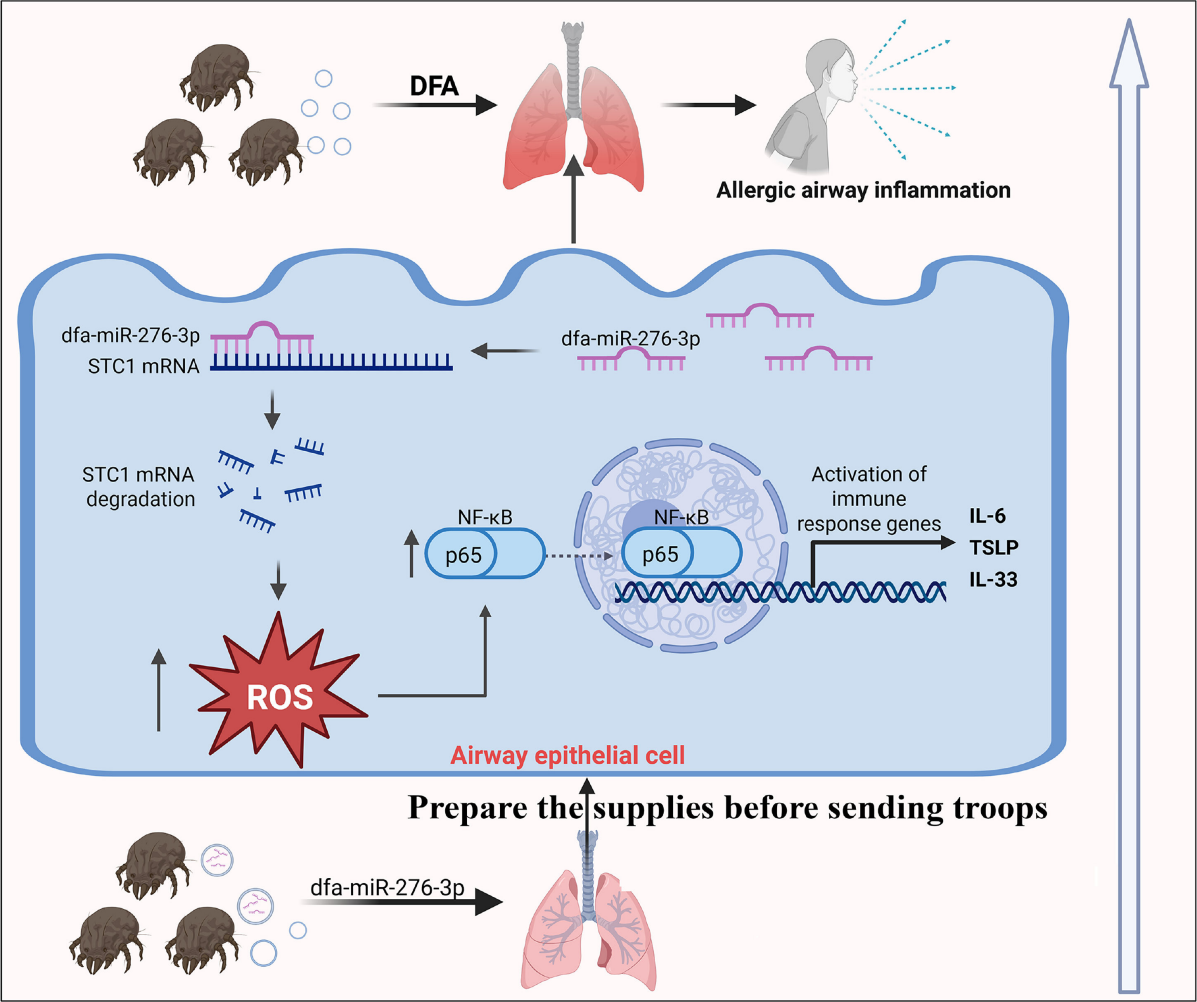
Flowchart for investigating the role of dfa-miR-276-3p in DFA-induced airway inflammation.

DFA, a major aeroallergen associated with allergic asthma (18), releases EVs containing dfa-miR-276-3p into household dust samples. Their nanoscale size facilitates inhalation in the airways. In this study, we confirmed the presence of DFA-derived dfa-miR-276-3p in residential environments, and this miRNA exerts cross-kingdom effects by suppressing STC1, which activates the ROS/NF-κB pathway and promotes the release of inflammatory cytokines, establishing a critical precursor for DFA-induced airway sensitization. This mechanism aligns with the ancient adage, “Prepare the supplies before sending troops,” underscoring the priming role of this miRNA in priming allergic inflammation (Fig. 7).

The discovery of EVs provides a valuable tool for studying cross-kingdom communication and the regulation of biological functions through their secretion and uptake (19). It has been demonstrated that hsa-miR-24-3p is packaged into EVs secreted by monocytes in response to *Candida albicans*, subsequently entering *C. albicans* to promote its growth (20). Our previous study showed that EVs derived from DFA effectively delivered dfa-miR-276-3p into BEAS-2B cells, in which dfa-miR-276-3p expression was observed (6). Based on these findings, we hypothesize that dfa-miR-276-3p is released upon the entry of EVs into airway epithelial cells, potentially playing a priming role in the sensitization process induced by DFA.

miRNAs are key post-transcriptional regulators of gene expression, and they are increasingly recognized for their roles in modulating inflammatory pathways (21). For instance, miR-34a and miR-34c enhance the production of chemokines and cytokines in keratinocytes by targeting LGR4 (22), while miR-146a-5p suppresses NLRP3 inflammasome activation through the TIRAP/NF-κB pathway (23). However, biological processes are governed by complex, multidimensional regulatory networks, and the limitations of individual miRNAs in driving specific outcomes are becoming increasingly evident (24). As one of the most abundant miRNAs in DFA (6), dfa-miR-276-3p primarily modulates the inflammatory microenvironment associated with DFA-induced allergic airway inflammation. Therefore, in our study, we first treated allergic animal and cell models with dfa-miR-276-3p prior to DFA stimulation, with the aim to investigate the role of dfa-miR-276-3p in DFA-sensitized airway inflammation.

miRNAs regulate gene expression by suppressing target genes (25). In this study, we identified STC1 as a direct target of dfa-miR-276-3p. STC1 is an anti-inflammatory glycoprotein (26) that inhibits thrombin signaling and protects against bleomycin-induced lung injury (27). Our study found that children allergic to DFA had lower serum STC1 levels than healthy controls, indicating its role in dfa-miR-276-3p-mediated airway inflammation. STC1 has been recently identified as a novel calcium-regulating protein that is closely associated with the development and progression of asthma (28, 29). In airway smooth muscle cells, STC1 was found to inhibit store-operated calcium entry by suppressing stromal interaction molecule 1 (STIM1), thereby preventing airway remodeling during the progression of bronchial asthma, and a deficiency of STC1 in asthmatic airways led to excessive activation of STIM1, enhancing smooth muscle contraction and resulting in airway hyper-responsiveness (30). This suggests that STC1 may function as a potential epithelial-derived relaxant factor. In addition, serum STC1 levels were reported to correlate with asthma control and lung function among asthmatic patients (30). In our study, we observed decreased STC1 expression levels in the serum of children with DFA-associated allergy and asthma. Taking these findings together, it is indicated that STC1 may have significant implications for the severity or activity of DFA-associated asthma and may serve as a potential biomarker for allergic asthma. Additionally, rhSTC1 was reported to reduce airway hyperresponsiveness and inflammation in murine asthma models (30) and alleviate oxidative stress and apoptosis when administered intratracheally (31). In the current study, we observed that rhSTC1 alleviated the inflammatory effects of dfa-miR-276-3p, demonstrating that this miRNA functions by regulating STC1. Mechanistically, STC1 suppresses inflammation by inhibiting ROS (15, 32), with higher STC1 levels leading to reduced ROS in airway epithelial cells and less fibrosis (33). Furthermore, overexpression of dfa-miR-276-3p resulted in increased ROS production in airway epithelial cells, suggesting that dfa-miR-276-3p promotes DFA-induced allergic inflammation through STC1-dependent dysregulation of ROS.

Previous studies have demonstrated that STC1 regulates NF-κB expression by suppressing ROS production, thereby reducing the levels of inflammatory cytokines in diabetic complications (34). The anti-inflammatory and neuroprotective effects of STC1 may involve the ROS/NF-κB pathway, while NF-κB activation may further enhance ROS generation, creating a feedback loop that amplifies inflammation (17). Our *in vitro* and *in vivo* experiments revealed that dfa-miR-276-3p modulated NF-κB activation by targeting STC1, influencing ROS generation. Upon stimulation by inflammatory cytokines or ROS, NF-κB phosphorylation and nuclear translocation drive the secretion of pro-inflammatory cytokines, such as IL-6, TSLP, and IL-33 (35, 36), confirming that dfa-miR-276-3p promotes inflammation in bronchial epithelial cells via the STC1-ROS/NF-κB axis, contributing to DFA sensitization.

The role of DFA-derived miRNAs has not been well understood in allergic reactions until now. Dfa-miR-276-3p, which is absent in mammals but highly expressed in arthropods (6), may contribute to hypersensitivity through cross-reactivity due to the similarity between allergens, such as tropomyosin found in shrimp and HDMs (37, 38). Considering its presence in DFA, mosquitoes (39), *Portunus trituberculatus* (40), and other insects, we hypothesize that miR-276 may act as a sensitizing factor, triggering cross-reactive hypersensitivity. In this study, we aimed to investigate dfa-miR-276-3p as a potential aggravator of allergies and as a modulator of the allergic microenvironment to better understand its sensitization mechanisms.

Detection of dfa-miR-276-3p in environmental samples suggests that this miRNA may enhance the allergenicity of DFA. Since avoiding allergens is crucial for preventing childhood allergic asthma (41), and previous studies primarily focused on DFA protein allergens (1, 18), our findings emphasize the role of miRNAs in DFA sensitization. Unlike proteins, miRNAs are resistant to heat degradation, and their stability may increase with longer length and higher GC content under mild heat (42, 43), such as during milk processing (44). However, ultra-high temperatures or prolonged heating may negate this stability (42). If synthetic miRNAs are susceptible to RNase degradation (45, 46), they may be stabilized through practical measures like higher drying temperatures, longer drying durations, or nuclease treatment to eliminate miRNA allergens. Additionally, DFA-derived EVs may enhance miRNA delivery (6). Due to their small size and slow sedimentation, EVs may remain airborne longer than dust particles (47). This necessitates that asthmatic children avoid allergens for extended periods after activities such as cleaning, along with implementing stricter isolation measures.

### Conclusions

This study identifies dfa-miR-276-3p as a novel mediator of airway inflammation through cross-kingdom regulation, providing new insights into the role of DFA-derived miRNAs in the development of allergic asthma. Although preliminary, our findings propose an alternative pathway for DFA sensitization that may have significant clinical implications for allergy prevention and treatment.

## Data Availability

All raw transcriptome sequencing data have been deposited in NCBI BioProject (accession number: PRJNA1023698). Children’s detailed demographic and clinical characteristics of the participants are demonstrated in the supporting file.

## SUPPLEMENTAL MATERIAL

Table S1 spectrum.01923-25-s0001.docx Sequences of primers used for PCR assay.

Table S2 spectrum.01923-25-s0002.docx Sequences of primers used for qPCR assay.

Table S3 spectrum.01923-25-s0003.docx Clinical information of 100 serum samples used in this research.

ASM does not own the copyrights to Supplemental Material that may be linked to, or accessed through, an article. The authors have granted ASM a non-exclusive, world-wide license to publish the Supplemental Material files. Please contact the corresponding author directly for reuse.
