# Supplementary material for: A novel role of Dermatophagoides farinae-derived miR-276-3p in aggravating mite-induced allergic airway inflammation
Source: Microbiol Spectr. 2025 Dec 22;14(2):e01923-25. doi: 10.1128/spectrum.01923-25 (PMC12889128; doi:10.1128/spectrum.01923-25)
Supplement: Table S1 — Sequences of primers used for PCR assay. [file spectrum.01923-25-s0001.docx]

**Table S1 Sequences of primers used for PCR assay**

| Primer | Forward (5’-3’) | Reverse (5’-3’) |
| --- | --- | --- |
| Outer-DFA | CAAGCGCTTGCCGTATCAATTCGGTTAACGTT | ATGTTGCGAATTTGGTCGTCGGCATTGTTGT |
| Inner-DFA | GGAATTGGATTTACGATCAC | GTATGGATAGCTTCTTTCTTC |
