# Supplementary material for: A novel role of Dermatophagoides farinae-derived miR-276-3p in aggravating mite-induced allergic airway inflammation
Source: Microbiol Spectr. 2025 Dec 22;14(2):e01923-25. doi: 10.1128/spectrum.01923-25 (PMC12889128; doi:10.1128/spectrum.01923-25)
Supplement: Table S2 — Sequences of primers used for qPCR assay. [file spectrum.01923-25-s0002.docx]

**Table S2 Sequences of primers used for qPCR assay**

| Primer | Forward (5’-3’) | Reverse (5’-3’) |
| --- | --- | --- |
| Human β-actin | ATTGCCGACAGGATGCAGAA | GCTGATCCACATCTGCTGGA |
| Human STC1 | GTGGCGGCTCAAAACTCAG | GTGGAGCACCTCCGAATGG |
| Mouse β-actin | CCACTGTCGAGTCGCGT | CCACGATGGAGGGGAATACAG |
| Mouse STC1 | ACACAGATGGGATGTACGACA | GGAAAGTCGAACACCTCCGA |
| EGFP | TGCTTCAGCCGCTACCC | AGTTCACCTTGATGCCGTTC |
| dfa-miR-276-3p | miR8009243 (Forward primer lot no.) | ssD089261711 (Reverse primer lot no.) |
| U6 | ssD0904071006 (Forward primer lot no.) |  |
| cel-miR-39-3p | ssD1083145002 (Forward primer lot no.) |  |
