# Supplementary material for: A novel role of Dermatophagoides farinae-derived miR-276-3p in aggravating mite-induced allergic airway inflammation
Source: Microbiol Spectr. 2025 Dec 22;14(2):e01923-25. doi: 10.1128/spectrum.01923-25 (PMC12889128; doi:10.1128/spectrum.01923-25)
Supplement: Table S3 — Clinical information of 100 serum samples used in this research. [file spectrum.01923-25-s0003.docx]

**Table S3 The clinical information of 100 sera samples in this research**

| Patients | Age(years) | Male/Female | Clinical history | ^#^IgE ((KUA/L) |
| --- | --- | --- | --- | --- |
| 1 | 9 | Female | NC | 0.04 |
| 2 | 13 | Female | NC | 0.02 |
| 3 | 3 | Female | NC | 0.02 |
| 4 | 5 | Male | NC | 0.04 |
| 5 | 8 | Female | NC | 0.02 |
| 6 | 3 | Male | NC | 0.01 |
| 7 | 8 | Female | NC | 0.04 |
| 8 | 10 | Male | NC | 0.16 |
| 9 | 8 | Female | NC | 0.03 |
| 10 | 1 | Female | NC | 0.02 |
| 11 | 3 | Female | NC | 0.03 |
| 12 | 5 | Male | NC | 0.07 |
| 13 | 8 | Female | NC | 0.05 |
| 14 | 8 | Male | NC | 0.09 |
| 15 | 7 | Female | NC | 0.02 |
| 16 | 8 | Female | NC | 0.04 |
| 17 | 7 | Female | NC | 0.01 |
| 18 | 2 | Female | NC | 0.02 |
| 19 | 7 | Female | NC | 0.22 |
| 20 | 3 | Female | NC | 0.01 |
| 21 | 7 | Female | NC | 0.10 |
| 22 | 6 | Male | NC | 0.17 |
| 23 | 4 | Female | NC | 0.01 |
| 24 | 4 | Male | NC | 0.03 |
| 25 | 10 | Male | NC | 0.03 |
| 26 | 1 | Female | NC | 0.07 |
| 27 | 4 | Male | NC | 0.02 |
| 28 | 8 | Female | NC | 0.04 |
| 29 | 7 | Female | NC | 0.25 |
| 30 | 7 | Female | NC | 0.02 |
| 31 | 7 | Female | NC | 0.02 |
| 32 | 5 | Male | NC | 0.08 |
| 33 | 8 | Female | NC | 0.07 |
| 34 | 6 | Female | NC | 0.03 |
| 35 | 6 | Male | NC | 0.02 |
| 36 | 1 | Female | NC | 0.03 |
| 37 | 3 | Male | NC | 0.03 |
| 38 | 6 | Female | NC | 0.21 |
| 39 | 3 | Female | NC | 0.03 |
| 40 | 5 | Male | NC | 0.21 |
| 41 | 1 | Female | NC | 0.02 |
| 42 | 13 | Male | NC | 0.05 |
| 43 | 7 | Male | NC | 0.04 |
| 44 | 5 | Male | NC | 0.12 |
| 45 | 5 | Male | NC | 0.03 |
| 46 | 3 | Female | NC | 0.02 |
| 47 | 6 | Male | NC | 0.03 |
| 48 | 14 | Male | NC | 0.03 |
| 49 | 4 | Male | NC | 0.02 |
| 50 | 8 | Male | NC | 0.07 |
| 51 | 11 | Female | BA | ＞100.00 |
| 52 | 3 | Male | BA | 91.2 |
| 53 | 10 | Male | AR | ＞100.00 |
| 54 | 7 | Male | AR | ＞100.00 |
| 55 | 4 | Female | BA | ＞100.00 |
| 56 | 4 | Female | BA | 82.3 |
| 57 | 8 | Male | BA | ＞100.00 |
| 58 | 9 | Male | BA | ＞100.00 |
| 59 | 7 | Male | BA | ＞100.00 |
| 60 | 4 | Male | AR | ＞100.00 |
| 61 | 11 | Male | BA | ＞100.00 |
| 62 | 5 | Male | AR | ＞100.00 |
| 63 | 3 | Male | AR | ＞100.00 |
| 64 | 3 | Male | BA | ＞100.00 |
| 65 | 13 | Female | AR | ＞100.00 |
| 66 | 3 | Male | BA | 87.2 |
| 67 | 5 | Female | BA | ＞100.00 |
| 68 | 4 | Male | BA | 82.1 |
| 69 | 6 | Male | BA | ＞100.00 |
| 70 | 4 | Female | AR | ＞100.00 |
| 71 | 5 | Male | AR | ＞100.00 |
| 72 | 5 | Male | AR | ＞100.00 |
| 73 | 3 | Female | BA | ＞100.00 |
| 74 | 6 | Male | BA | ＞100.00 |
| 75 | 11 | Male | BA | ＞100.00 |
| 76 | 13 | Male | AR | ＞100.00 |
| 77 | 8 | Female | AR | ＞100.00 |
| 78 | 8 | Male | BA | ＞100.00 |
| 79 | 9 | Male | BA | ＞100.00 |
| 80 | 7 | Male | BA | ＞100.00 |
| 81 | 7 | Female | BA | ＞100.00 |
| 82 | 4 | Male | BA | ＞100.00 |
| 83 | 4 | Male | AR | ＞100.00 |
| 84 | 9 | Female | BA | ＞100.00 |
| 85 | 8 | Male | AR | ＞100.00 |
| 86 | 6 | Female | AR | ＞100.00 |
| 87 | 6 | Male | AR | ＞100.00 |
| 88 | 11 | Male | AR | ＞100.00 |
| 89 | 14 | Male | AR | ＞100.00 |
| 90 | 5 | Female | AR | ＞100.00 |
| 91 | 8 | Female | BA | ＞100.00 |
| 92 | 10 | Male | AR | ＞100.00 |
| 93 | 10 | Male | BA | ＞100.00 |
| 94 | 5 | Male | AR | ＞100.00 |
| 95 | 8 | Female | BA | ＞100.00 |
| 96 | 6 | Female | AR | ＞100.00 |
| 97 | 8 | Male | BA | ＞100.00 |
| 98 | 5 | Male | BA | ＞100.00 |
| 99 | 5 | Male | AR | ＞100.00 |
| 100 | 11 | Male | AR | ＞100.00 |

BA: bronchial asthma; AR: allergic rhinitis; #IgE ((KUA/L): IgE specific to DFA
